# Analysis of free radical production capacity in mouse faeces and its possible application in evaluating the intestinal environment: a pilot study

**DOI:** 10.1038/s41598-019-56004-x

**Published:** 2019-12-20

**Authors:** Yoshihisa Wakita, Asako Saiki, Hirotaka Kaneda, Shuichi Segawa, Youichi Tsuchiya, Hiromi Kameya, Susumu Okamoto

**Affiliations:** 1 Frontier Laboratories for Value Creation, SAPPORO HOLDINGS LTD., Yaizu, Shizuoka 425-0013 Japan; 2 Food Research Institute, National Agriculture and Food Research Organization, Tsukuba, 305-8642 Japan

**Keywords:** Microbiology techniques, Environmental microbiology, Microbiome

## Abstract

Complex interplay between the intestinal environment and the host has attracted considerable attention and has been well studied with respect to the gut microbiome and metabolome. Oxygen free radicals such as superoxide and the hydroxyl radical (^•^OH) are generated during normal cellular metabolism. They are toxic to both eukaryotic and prokaryotic cells and might thus affect intestinal homeostasis. However, the effect of oxygen free radicals on the intestinal environment has not been widely studied. Herein, we applied electron spin resonance spectroscopy with spin trapping reagents to evaluate oxygen free radical production capacity in the intestinal lumen and the faeces of mice. ^•^OH was generated in faeces and lumens of the small and large intestines. There were no remarkable differences in ^•^OH levels between faeces and the large intestine, suggesting that faeces can be used as alternative samples to estimate the ^•^OH production capacity in the colonic contents. We then compared free radical levels in faecal samples among five different mouse strains (ddY, ICR, C57BL/6, C3H/HeJ, and BALB/c) and found that strain ddY had considerably higher levels than the other four strains. In addition, strain ddY was more susceptible to dextran sulphate sodium-induced colitis. These differences were possibly related to the relative abundance of the gut bacterial group *Candidatus* Arthromitus, which is known to modulate the host immune response. From these results, we suggest that the production capacity of oxygen free radicals in mouse faeces is associated with intestinal homeostasis.

## Introduction

The intestinal environment is complex and dynamic and is composed of constituents of the diet, digestion products, secretions from the host, the microbiome, and microbial metabolites. Recent human cohort studies have revealed a significant influence of the intestinal microbiome on the host’s health. Inflammatory bowel disease (IBD)^1^, obesity^2^, type 2 diabetes^3^, and atherosclerosis^4^ have been all associated with changes in the intestinal microbiome. An increase in the Firmicutes/Bacteroidetes^5^ ratio has been implicated in obesity, whereas dysbiosis might contribute to the pathogenesis of both intestinal and extra-intestinal disorders^6–8^. However, there are significant inter-individual differences in the intestinal microbiome, even in patients with the same disease^9^, making it difficult to draw conclusions. It is possible that beneficial or toxic substances produced by specific bacteria or by the host might have a critical role in modulating host health.

Oxygen free radicals, such as superoxide (O_2_^•−^) and the hydroxyl radical (^•^OH), are generated by aerobic respiration in bacteria^10^ and are toxic to both eukaryotic and prokaryotic cells. As the lumen of the large intestine is devoid of oxygen, obligate anaerobes, such as *Bacteroides*, *Clostridium*, and *Ruminococcus*, are major inhabitants of this niche. However, the numbers of oxygen-tolerant facultative anaerobes such as members of Enterobacteriaceae and Enterococcaceae increase during dysbiosis^11,12^. Moreover, the existence of an intraluminal oxygen gradient has been recently reported^13^. Based on these observations, we considered the possibility that free radicals are generated within the lumen of the large intestine and that they influence host health. In fact, Huycke *et al*.^14^ have reported that ^•^OH and O_2_^•−^ can be observed in the colon contents of rats inoculated with specific strains of *Enterococcus faecalis*. These findings warrant further investigation to elucidate the interaction between the intestinal environment and host health based on the role of oxygen free radicals.

Electron spin resonance (ESR) spectroscopy is a useful technique that can detect, identify, and quantify various free radicals and has been successfully used to analyse oxygen free radicals in a variety of biological samples. O_2_^•−^ and ^•^OH are highly oxidizing species with a reduction potential of ~1.6 V and ~2.6–2.7 V, respectively^15^. These oxygen free radicals instantly react with almost all biomolecules, and therefore they can only be trapped by a spin trap, provided that the spin trap is in close proximity to the radicals at the exact moment when they are generated. In this study, we applied ESR with spin trapping reagents to assess free radical production capacity in the intestinal lumen and faeces of mice. We found significant differences in faecal free radical production capability among mouse strains with different genetic backgrounds. A possible relationship between this faecal free radical production capacity and the intestinal microbiome and/or intestinal homeostasis is also discussed.

## Results

### Free radical production capacity in the small and large intestines and faeces

**Figure 1:**
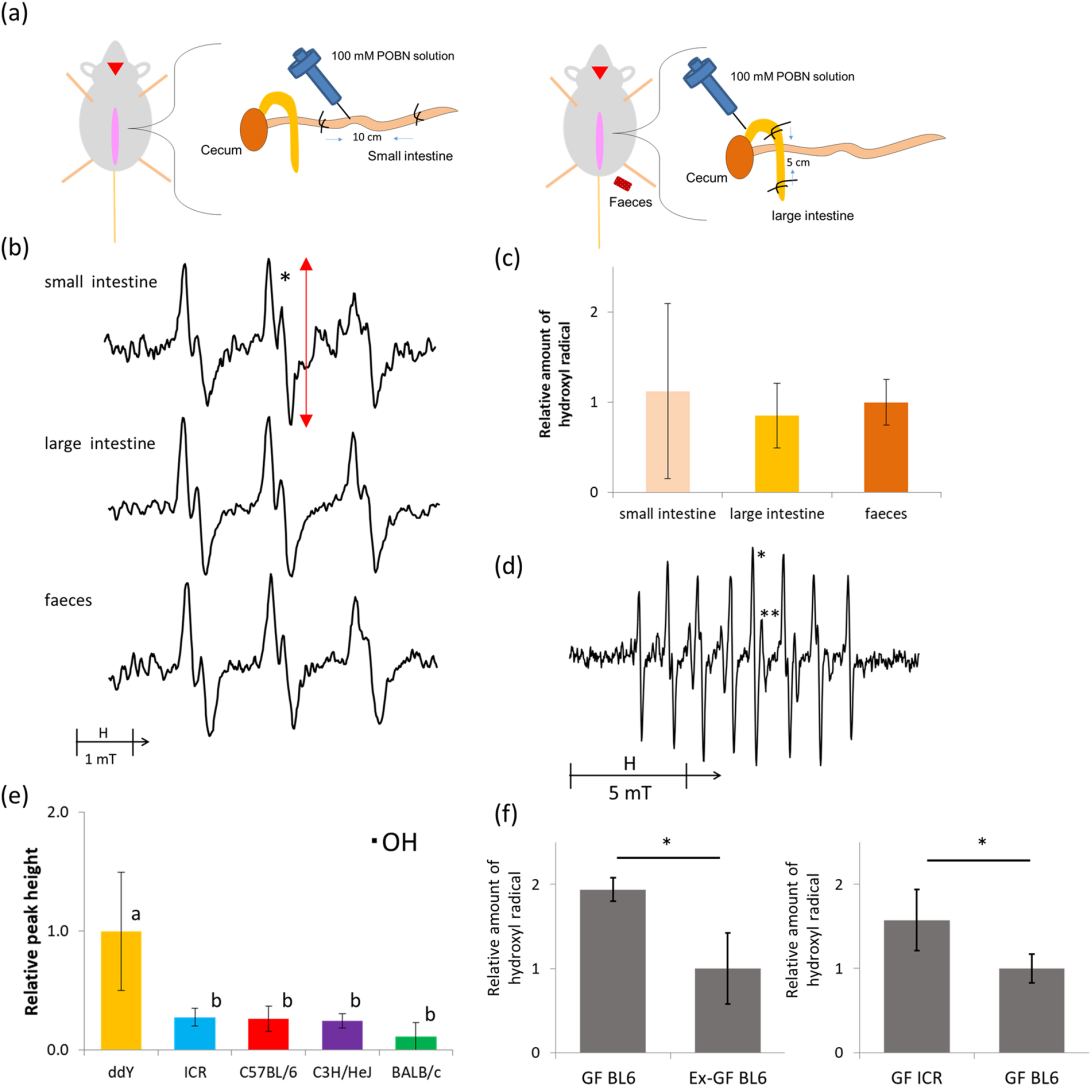
Oxygen free radical production capacity in faeces and the small and large intestines. (**a**) Schematic diagram of ligated intestinal loop assay. ddY mice were anesthetized with isoflurane gas. After opening the abdomen, both ends of the large or small intestine were bound with surgical thread, and POBN solution was injected. The contents were collected and centrifuged, and the supernatants were analysed by electron spin resonance (ESR). Faeces were reacted with POBN solution and analysed similarly. (**b**) ESR spectra from faeces and the small and large intestines. The center line (from the bottom to the top of the peak) marked with an asterisk (*) was used to calculate the relative amounts of ^•^OH. (**c**) Relative amounts of ^•^OH. Data are shown as mean values ± SD. N = 6–7 per group. One-way ANOVA showed no significant differences among ^•^OH levels in the faeces, small intestine, and large intestine. (**d**) A typical ESR spectrum from faeces obtained with the spin-trapping agent CYPMPO. The peak-to-peak intensities of the line marked with an asterisk (*) and double asterisk (**) were used to calculate relative amounts of ^•^OH and O_2_^•−^, respectively. (**e**) Relative amounts of ^•^OH in different mouse strains. Data are shown as mean values ± SD (n = 6 per group). There were significant differences for ddY vs. ICR, C57BL/6, C3H/HeJ, and BALB/c. *p < 0.001 (vs. ddY). (**f**) Relative levels of ^•^OH in germ free (GF) and ex-germ-free (Ex-GF) mice Ex-GF mice were inoculated with faeces obtained from SPF C57BL/6 mice. Data are shown as mean values ± SD (n = 10 per group). *p < 0.001.

To evaluate the capacity of oxygen free radical production in the lumens of the large and small intestines, we performed a ligated intestinal loop experiment (Fig. 1a) and oxygen free radicals were detected by the ESR spin-trapping technique (Fig. 1b). At the same time, we analysed free radicals in faecal samples. Alpha-(4-pyridyl *N*-oxide)-*N*-*tert*-butyl nitrone (POBN) was used in this study, as the spin adduct from this spin trap is stable in biological environments^16^, and a sharp signal was observed. As expected, a signal from the POBN spin adduct was observed in all three sample sets. From the hyperfine coupling constant of the spectra (*a*_N_ = 1.52 mT, *a*_H_ = 0.22 mT), this POBN adduct was identified as POBN-^•^OH with reference to a previous report^17^, demonstrating the presence of ^•^OH production capacity in both the intestinal lumen and faeces. ^•^OH levels were similar in the large intestine and faeces (Fig. 1c), suggesting that ^•^OH production capacity in faeces could be used to predict that in the large intestinal lumen.

### Oxygen free radical production capacity in mouse faeces from different strains

The genetic background of the host may affect free radical production capacity in the intestinal environment. Thus, we analysed this in the faeces of five different mouse strains from the same breeder. In this analysis, we used 5-(2,2-dimethyl-1,3-propoxy cyclophosphoryl)-5-Methyl-1-pyrroline *N*-oxide (CYPMPO) as a spin-trap agent, because it is more suitable for faecal samples, and more importantly, it enables precise discrimination between ^•^OH and O_2_^•−^. A typical ESR spectrum obtained with CYPMPO is shown Fig. 1d. By calculation of the hyperfine coupling constant, ^•^OH (*a*_N_ = 1.37, 1.35 mT; *a*_H_ = 1.36, 1.23 mT; *a*_P_ = 4.87, 4.68 mT) and O_2_^•−^ (*a*_N_ = 1.27, 1.27 mT; *a*_H_ = 1.11, 1.06 mT; *a*_P_ = 5.24, 5.09 mT) were unambiguously identified with reference to a previous report^18^. However, O_2_^•−^ levels were often very low, and thus, we used only ^•^OH for further analysis. Surprisingly, ddY mice had significantly higher ^•^OH levels than the other four strains (*p* < 0.001). In contrast, BALB/c mice exhibited the lowest free radical levels, although there were no statistically significant differences (Fig. 1e).

### Comparison of faecal oxygen free radical levels in germ-free and ex-germ-free mice

It is possible that free radical levels in the intestinal environment are influenced by the gut microbiome or host genetic background. To explore this possibility, we evaluated oxygen free radical production capacity in the faeces of germ-free (GF) and ex-germ-free (Ex-GF) mice (Fig. 1f). The ^•^OH levels in GF-C57BL/6 mice were higher than those in Ex-GF C57BL/6 mice inoculated with faeces from SPF-C57BL/6, indicating that the intestinal microbiome negatively affects ^•^OH levels in this mouse strain. Furthermore, ^•^OH levels in GF-ICR mice were higher than those in GF-C57BL/6 mice. Therefore, the host genetic background might also determine faecal ^•^OH production capability.

### Faecal microbiome analysis

**Figure 2:**
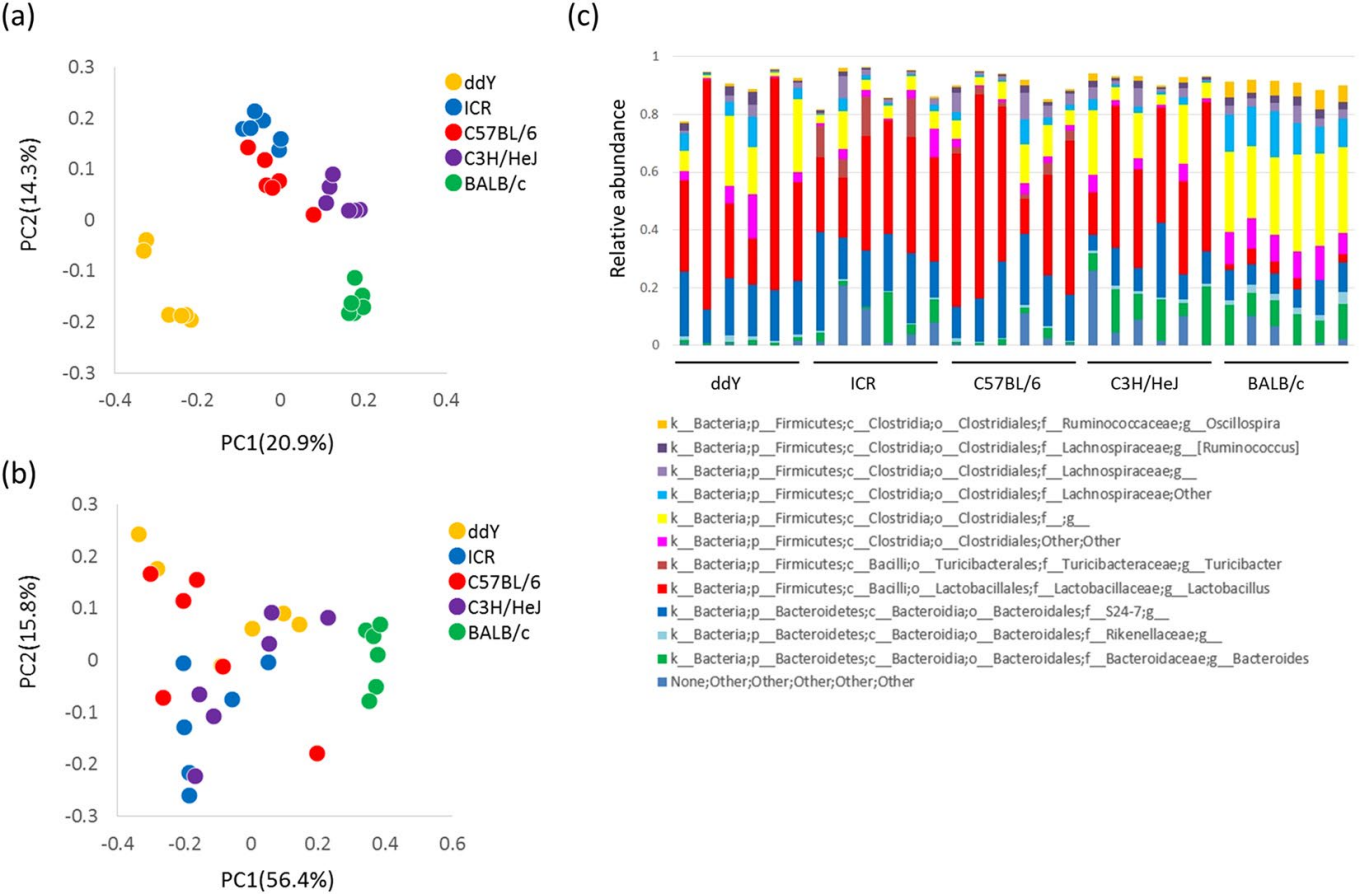
Structure of intestinal microbiome in different mouse strains. Amplicon sequencing was performed using the V1/V2 regions of the 16S rRNA gene, followed by analysis using the QIIME pipeline. (**a**) Unweighted unifrac analysis of intestinal microbiome. (**b**) Weighted unifrac analysis of intestinal microbiome. (**c**) Relative abundance of intestinal microbiota at the genus level. Genera for which the abundance was greater than 2% are shown.

**Figure 3:**
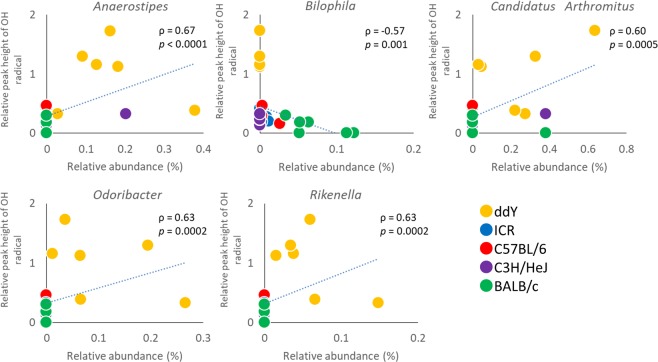
Correlation between relative abundance of genera and relative peak height of ^•^OH. Correlation between relative abundance of 24 genera (Fig. S1) and relative peak heights of ^•^OH were evaluated by Spearman’s rank correlation coefficient. A probability value of less than 0.0021 (0.05/24) was considered statistically significant. The genera for which the probability value was less than 0.0021 are shown.

The gut microbiota is likely a key determinant of oxygen free radical production capacity in the mouse intestinal environment. To gain insight into this process, we carried out faecal microbiome analysis. A total of 1,055,363 reads (23,142–57,527 reads per sample) were obtained by sequencing and selection using the QIIME pipeline. Initially, the overall structure of the microbiome was evaluated using unweighted and weighted unifrac analysis (Fig. 2a,b). The unweighted unifrac analysis revealed differences between mouse strains. The microbiomes of ddY or BALB/c mice particularly differed from those of the other three mouse strains. Weighted unifrac analysis showed differences in microbial structure between BLAB/c and other mouse strains. Subsequently, differences in microbial composition were taxonomically evaluated at the phylum and genus levels (Fig. 2c). A total of 10 phyla and 73 genera were taxonomically identified. Although there were no clear differences in the microbial composition between the mouse strains at the phylum level, large differences were observed at the genus level (Fig. 2c). Compared to that in the other four mouse strains, BALB/c mice showed a lower relative abundance of *Lactobacillus* (Figs. 2c and S1). The genera in which significant differences were observed are shown in Fig. S1. The genera that correlated with ^•^OH production were *Anaerostipes*, *Bilophila*, *Candidatus* Arthromitus, *Odoribacter*, and *Rikenella* (*p* < 0.0021; Fig. 3).

### Susceptibility to dextran sulphate sodium (DSS)-induced colitis

**Figure 4:**
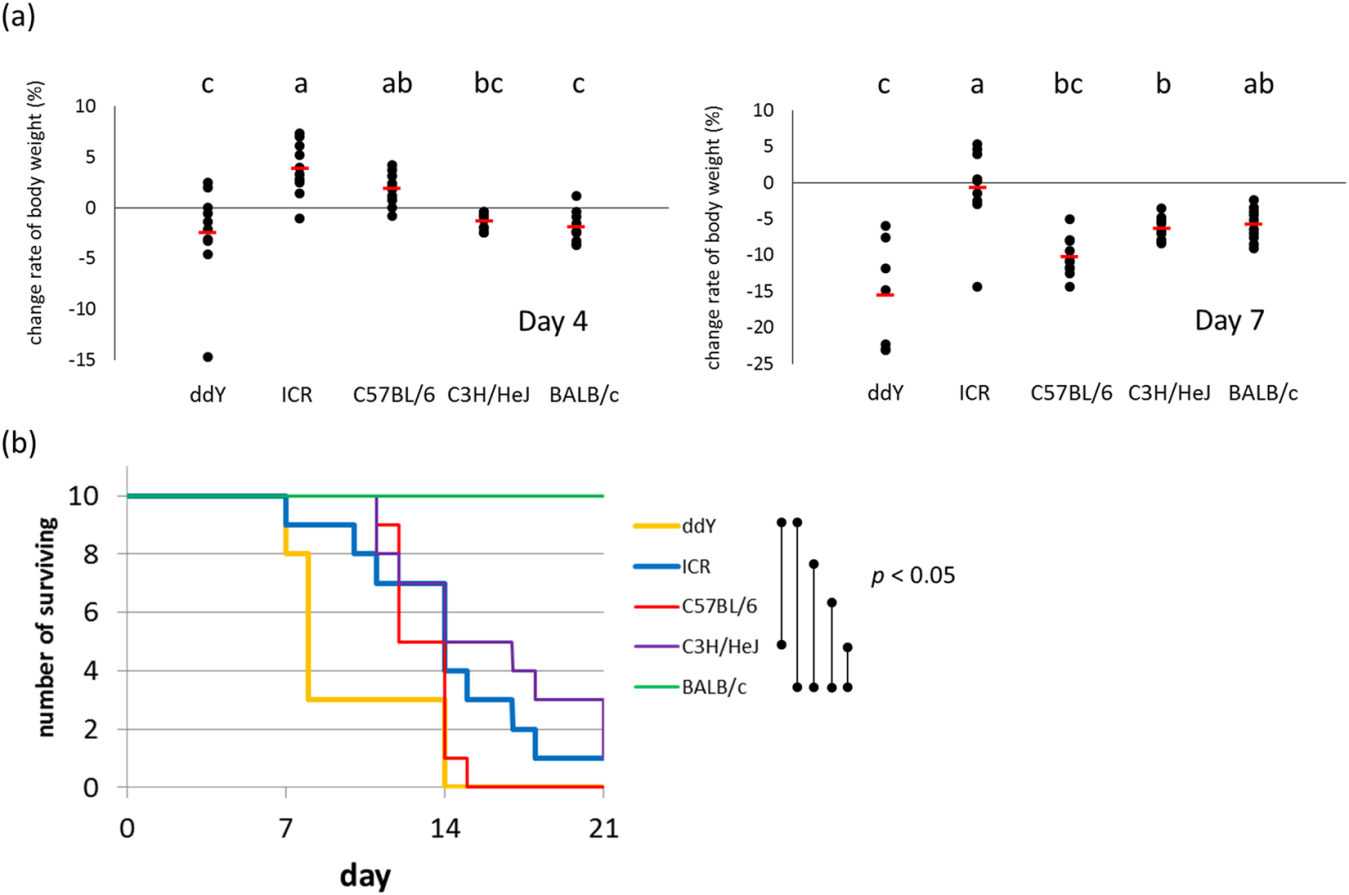
A comparison of responses to dextran sulphate sodium (DSS)-induced colitis among five different mouse strains. (**a**) Change in body weight on days 4 and 7. (**b**) Cumulative survival rate. Seven-week-old mice were used (n = 10 per group). Mice were allowed free access to 4% DSS water.

The experiment described previously revealed that the mouse strain ddY exhibited significantly higher levels of faecal free radicals. This might reflect perturbations to intestinal homeostasis. To explore this possibility, we compared susceptibilities to DSS-induced colitis among five mouse strains used previously. On day 4, the body weight of ddY mice was significantly lower than that of ICR and C57BL/6 mice (Fig. 4a). Further, on day 7, the body weight of ddY mice was significantly lower than that of the other four strains, indicating greater susceptibility of ddY mice to DSS-induced colitis. Furthermore, the survival rate was significantly lower in ddY mice, again demonstrating the marked susceptibility of this strain (Fig. 4b). Interestingly, BALB/c mice, which had the lowest radical levels, were highly resistant to DSS-induced colitis.

## Discussion

Here, we developed a simple method to evaluate oxygen free radical production capacity in the intestinal environment using the ESR spin-trapping technique. Babbs^19^ and Erhardt *et al*.^20^ have reported methods to quantify ^•^OH production capacity in faeces using DMSO. The reaction times of faeces and DMSO were 5 h and 16 h, respectively. Besides, methane sulfinic acid, which is a byproduct of the reaction between DMSO and ^•^OH, have been routinely analyzed as a proxy of the ^•^OH production capacity in faeces. In comparison with these methods, ESR spin-trapping technique is capable of detecting free radicals in a short time and in a more immediate way. In this study, we firstly compare the ^•^OH production capacities in faeces and colonic contents. Our results suggest that faeces can be used as alternative samples to estimate the ^•^OH production capacity in the colonic contents.

Oxidative stress in intestinal epithelial cells has been implicated in the initiation of colonic diseases such as IBD^21^ and colon cancer^22^. Many studies have focused on the host inflammatory response, which results in increased oxidative stress^23,24^; however, little attention has been paid to oxygen free radicals in the gastrointestinal tract, probably because of the low O_2_ concentration in the intestinal lumen and separation of epithelial cells and colonic contents by mucus layer. Nonetheless, ^•^OH could be generated from H_2_O_2_ by the Fenton reaction, and it has been reported that certain intestinal bacteria such as *Lactobacillus*, *Enterococcus*, and *Streptococcus* have the ability to produce H_2_O_2_^25^. In addition, a report that no mucus layer separates colonic contents from epithelium in the proximal colon has been reported^26^. We also observed similar results (Fig. S2). Thus, it is not unreasonable to consider the oxygen exposure of colonic contents and free radical generation in the colon. Oxygen free radicals in the intestinal environment can have a negative influence on intestinal homeostasis; specifically, O_2_^•−^ is reportedly produced in the colon of rats inoculated with a specific strain of *Enterococcus faecalis*, and this radical has been implicated in colon cancer^14^. In this regard, quantitative analysis of both ^•^OH and O_2_^•−^ production capacity in the intestinal environment would be valuable; in this study, both were found to be generated in faeces, whereas ^•^OH was the predominant species in normal faecal samples.

The lower ^•^OH levels in Ex-GF-mice compared to those in GF-mice implied the role of the intestinal microbiome in the elimination of oxygen free radicals. In addition, the host genetic background was also found to affect ^•^OH levels. These results suggest that a complex mechanism involving both the intestinal microbiome and host genetic background determines the levels of oxygen free radicals. Sex- and strain-dependent differences in the intestinal microbiome in mice have been reported^27^. In addition, the association between host genetics and heritable bacterial taxa in the human intestinal microbiome has been reported^28^. Therefore, it is possible that the host genetic background affects ^•^OH levels in the intestinal environment, which in turn influences the composition of the intestinal microbiome.

DSS-induced colitis in mice has widely been used as an animal model of IBD^29,30^, in which the intestinal microbiome might play a role in the induction of colitis. Recent studies have shown the interaction between specific bacteria and the host immune response^31–34^. Our results show that susceptibility to DSS-induced colitis varies significantly between mouse strains. Moreover, mouse strains showing higher ^•^OH levels tended to be more susceptible to IBD. Our genus-level classification of the intestinal microbiome showed that ^•^OH levels were correlated with the relative abundance of several genera, and particularly *Candidatus* Arthromitus, a segmented filamentous bacterial group that is oxygen-tolerant^35^ and known to activate Th17 cells^34^. Together, these findings suggest that certain bacteria modulate intestinal homeostasis via free radical production, or that free radicals generated by the host metabolism influence the survival of certain bacteria. However, it is necessary to verify whether the free radical production capacity is linked to the onset of colitis, and test if radical scavengers could influence the intestinal microbiome or the outcomes of colitis.

BALB/c mice, which showed the lowest faecal ^•^OH levels and a marked resistance to DSS-induced colitis, exhibited a low relative abundance of the genus *Lactobacillus* compared to that in the other four mouse strains. Intestinal inflammation can lead to dysbiosis, which is characterized by a marked decrease in the occupancy of obligate anaerobes and an increased relative abundance of facultative anaerobes^36^. Although the beneficial effects of specific strains of *Lactobacillus* have been suggested^37–39^, an increase in commensal facultative anaerobes including *Lactobacillus* might reflect enhanced oxidative stress in the lumen. The reported increase in the abundance of facultative anaerobes such as *Lactobacillus* during aging in humans^40^ is probably related to this notion. Similar changes have been observed in an accelerated mouse model of aging^41^. Thus, understanding the complex interplay between free radicals and facultative anaerobes in the intestinal environment, intestinal homeostasis, and aging is important.

In conclusion, rapid and straightforward experimental techniques to evaluate oxygen free radical production capacity in mouse faeces were established, and we propose their possible application to evaluating the intestinal environment. Although this study did not show direct evidence of free radical production in the intestinal environment, the association between free radical production capacity in faeces and the intestinal microbiome or physiology was significant. Further, it provides a novel perspective in the field of intestinal microbiology. In the future, research combining our free radical analysis with microbiome, metagenome, and metabolome analyses to evaluate the relationships between the intestinal environment and host health status might lead to a better understanding of the complex interplay between the intestinal microbiome and the host.

## Materials and Methods

### Mice

Six- to seven-week-old male ddY, ICR, C57BL/6, C3H/HeJ, and BALB/c mice were purchased from Japan SLC Inc. (Shizuoka, Japan). The mice were bred in Frontier Laboratories for Value Creation, Sapporo Holdings Ltd., Shizuoka, Japan. They were housed at 4–5 per cage (depth 26, width 18, and height 13 cm), and given water and commercial CRF-1 pellets (CLEA Japan, Inc.) *ad libitum*. After >1 week of preliminary breeding, the mice were used for experiments. In addition, 6–7-week-old male GF-ICR and GF-C57BL/6 mice were purchased from CLEA Japan, Inc. (Tokyo, Japan). GF-mice were housed in flexible film plastic isolators (CLEA Japan, Inc.) and given sterilized water and sterilized CRF-1 pellets *ad libitum*. The diet was sterilized using an autoclave (121 °C, 60 min). After 1 week of preliminary breeding, Ex-GF mice were inoculated with 0.2 mL of a 10^−1^ suspension of faeces obtained from SPF C57BL/6 mice in the stomach using a metal catheter. The protocols were approved by the Sapporo Holdings Animal Use Committee (permit number: 2015–016), and all the experimental procedures were performed according to the guidelines of the Animal Care Committee of Sapporo Holdings Ltd. and were in accordance with the Guide for the Care and Use of Laboratory Animals published by the National Academies Press.

### Free radical production capacity in faeces and the intestine

Faeces were collected from 8-week-old-mice and stored on crushed ice until use (approximately 1–3 h). POBN (Tokyo Chemical Industry Co., Ltd., Tokyo, Japan) was used for spin trapping. Faecal samples (approximately 200 mg) were diluted nine-fold with 100 mM POBN solution (Dulbecco’s phosphate buffered saline, 50 mM EDTA, pH 7.4) and mixed for 1 min with a spatula. After 15 s of weak vortexing, samples were left to sit at room temperature (approximately 25 °C) for 30 min. The samples were centrifuged at 12,000 × *g* for 2 min at 25 °C, and 500 μL of the supernatant was transferred into a flat quartz ESR cell and measured at 25 °C (Keycom X10SA X-band spectrometer; Keycom Corp., Tokyo, Japan). The measurement conditions were as follows: field modulation, 100 kHz; microwave power, 16 mW; field scan width and rate, 16 mT/60 s; time constant, 0.03 s.

To analyse intestinal free radicals, the ligated intestinal loop technique was applied. Mice were anesthetized with isoflurane gas. After opening the abdomen, both ends of the large intestine (2–7 cm from cecum) or small intestine (5–15 cm from cecum) were bound with surgical thread, and 700 μL (for large intestine) or 800 μL (for small intestine) of 100 mM POBN solution was injected using a 22 G intravenous cannula. Thirty minutes after injection, intestinal contents were collected and centrifuged at 12,000 × *g* for 2 min at 25 °C. The supernatants were analysed by the aforementioned method, whereas precipitates were freeze-dried and weighed. The peak height was normalized by the signal intensity of Mn marker, and corrected by the weight of precipitate. Mice used for the analysis of the large intestine were the same populations as those used for the analysis of faeces. In contrast, mice used for the analysis of the small intestine belonged to separate populations.

### Analysis of free radicals in faeces of different mouse strains, GF-mice, and Ex-GF mice

Faeces were collected from 8-week-old male ddY, ICR, C57BL/6, C3H/HeJ, and BALB/c mice, which were used for both free radical and microbiome analyses. Faeces were collected from 7–8-week old male GF-ICR and GF-C57BL/6 mice. Faeces from Ex-GF C57BL/6 mice were collected after 10 days of faecal transplantation. The samples were stored on crushed ice until use. CYPMPO (Shidai System, Tokyo, Japan) was used for spin trapping. Samples (approximately 100 mg) were diluted nine-fold with 100 mM CYPMPO solution (100 mM tris, 50 mM EDTA, pH 8.0) and mixed for 1 min with a spatula. After 15 s of weak vortexing, the samples were left to sit at room temperature (approximately 25 °C) for 30 min. The samples were centrifuged at 3,000 × *g* for 1 min at 25 °C, and approximately 300 μL of the supernatant was transferred to a flat disposable ESR cell (Radical Research Inc., Hino, Japan). Measurements were performed at 25 °C using a Bruker EMX-plus X-band spectrometer (Bruker Biospin, Billerica, Massachusetts, USA). The measurement conditions were as follows: field modulation, 100 kHz; resonance field, 342–362 mT; field modulation width, 0.1 mT; microwave power, 6 mW; time constant, 0.1 s; scan time, 10 times. The peak height was normalized by the signal intensity of Mn marker.

### Microbiome analysis

Fresh faecal samples (approximately 100 mg) were washed twice with 1 mL Dulbecco’s phosphate buffered saline, stored at −80 °C, and used for the analysis of the intestinal microbiome. Bacterial DNA was isolated using the method described by Matsuki *et al*.^42^, with some modifications. Briefly, the bacterial suspension was treated with lysis buffer at 70 °C for 10 min in a water bath and vortexed vigorously for 40 s at 6.0 m/s.

Primers for the amplification of the V1 and V2 regions of the 16S rRNA gene, reported by Kim *et al*.^43^, were used with some modifications^44^ to allow for the use of the Ion PGM sequencer (Life Technologies, Carlsbad, CA, USA). PCR was performed in a reaction volume of 25 μL. Each reaction mixture contained 22.5 μL of platinum PCR mix, 2 μL of template DNA (~4 ng), and 0.5 μL of 10 μM primer mix. The amplification reaction was carried out in the Veriti Thermal Cycler (Applied Biosystems, Foster City, CA, USA) using the following program: 3 min at 94 °C, followed by 25 cycles of 30 s, each at 94 °C, 45 s at 55 °C, and 1 min at 68 °C. After each reaction, the mixture was purified using a PureLink Quick PCR Purification Kit (Invitrogen, Carlsbad, CA, USA); the concentration of each purified sample was measured using a Qubit 2.0 fluorometer (Life Technologies). Purified samples were mixed at equal concentrations. The mixed samples were visualized by electrophoresis on 2% agarose gels and purified by gel extraction using the FastGene Gel/PCR Extraction Kit (Nippon Gene Co. Ltd., Tokyo, Japan). Subsequently, emulsion PCR and sequencing were carried out using the Ion PGM sequencing system (Life Technologies).

After sequencing, the obtained reads were analysed by the QIIME pipeline^45^ (http://qiime.org/) for an assignment of taxonomic classification. The reads that included precise primer sequences were selected, and those with an average quality value >20 were used for further analysis. The reads were grouped into operational taxonomic units (OTUs), with a sequence identity threshold of 97%, and chimeric OTUs were removed using ChimeraSlayer. Unifrac distance analysis was performed, and the proportions of intestinal microbiota at the genus level were determined based on the RDP classifier.

### DSS-induced colitis

Seven-week-old ddY, ICR, C57BL/6, C3H/HeJ, and BALB/c mice were used (n = 10 per group). DSS, with a molecular weight of 25,000 (Tokyo Chemical Industry, Tokyo, Japan), was dissolved in tap water to obtain a 4% DSS solution. Mice were allowed free access to the 4% DSS solution. The weight and cumulative survival rate of the mice were then evaluated.

### Statistical analysis

One-way analysis of variance with a *post-hoc* Turkey HSD test was performed for the statistical evaluation of the levels of free radicals and the intestinal microbiome composition. The Spearman’s rank correlation coefficient was used to analyse correlations between intestinal bacteria and free radical production. A probability value of less than 0.05 ÷ number of tests was considered statistically significant. For DSS-induced colitis experiments, the statistical evaluation of differences in survival rates was performed by the generalized Wilcoxon test with Holm-correction by 10 pairs. A probability value of less than 0.05 was considered statistically significant.

## Supplementary information

Supplementary information

## Data Availability

All sequence data were deposited in the DDBJ sequence read archive database under the accession number DRA008158.
