# Supplementary material for: Analysis of free radical production capacity in mouse faeces and its possible application in evaluating the intestinal environment: a pilot study
Source: Sci Rep. 2019 Dec 20;9:19533. doi: 10.1038/s41598-019-56004-x (PMC6925209; doi:10.1038/s41598-019-56004-x)
Supplement: Supplementary file 1 — Supplementary information [file 41598_2019_56004_MOESM1_ESM.pdf]

Supplementary Information

Analysis of free radical production capacity in mouse faeces and its possible application in evaluating the intestinal environment: a pilot study

Yoshihisa Wakita[1*], Asako Saiki[1], Hirotaka Kaneda[1], Shuichi Segawa[1], Youichi Tsuchiya[1], Hiromi Kameya[2], Susumu Okamoto[2]

[1] Frontier Laboratories for Value Creation, SAPPORO HOLDINGS LTD., Yaizu, Shizuoka 425-0013, Japan;
[2] Food Research Institute, National Agriculture and Food Research Organization, Tsukuba 305-8642, Japan

[*]Corresponding author

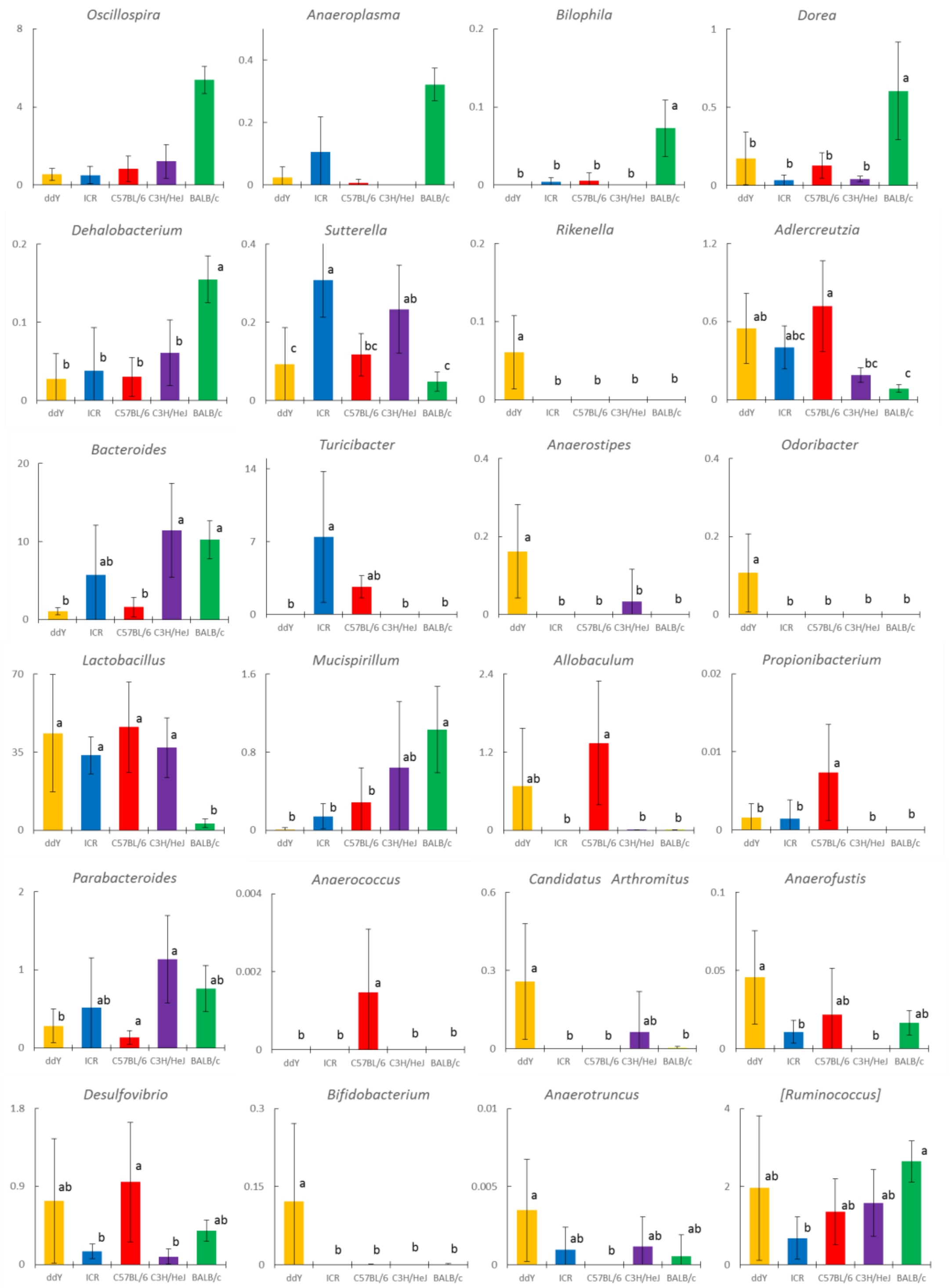

Figure S1: Differences in genera of faecal microbiomes between mouse strains.

Data are shown mean values ± SD (n = 6 per group.).

(a) (b)

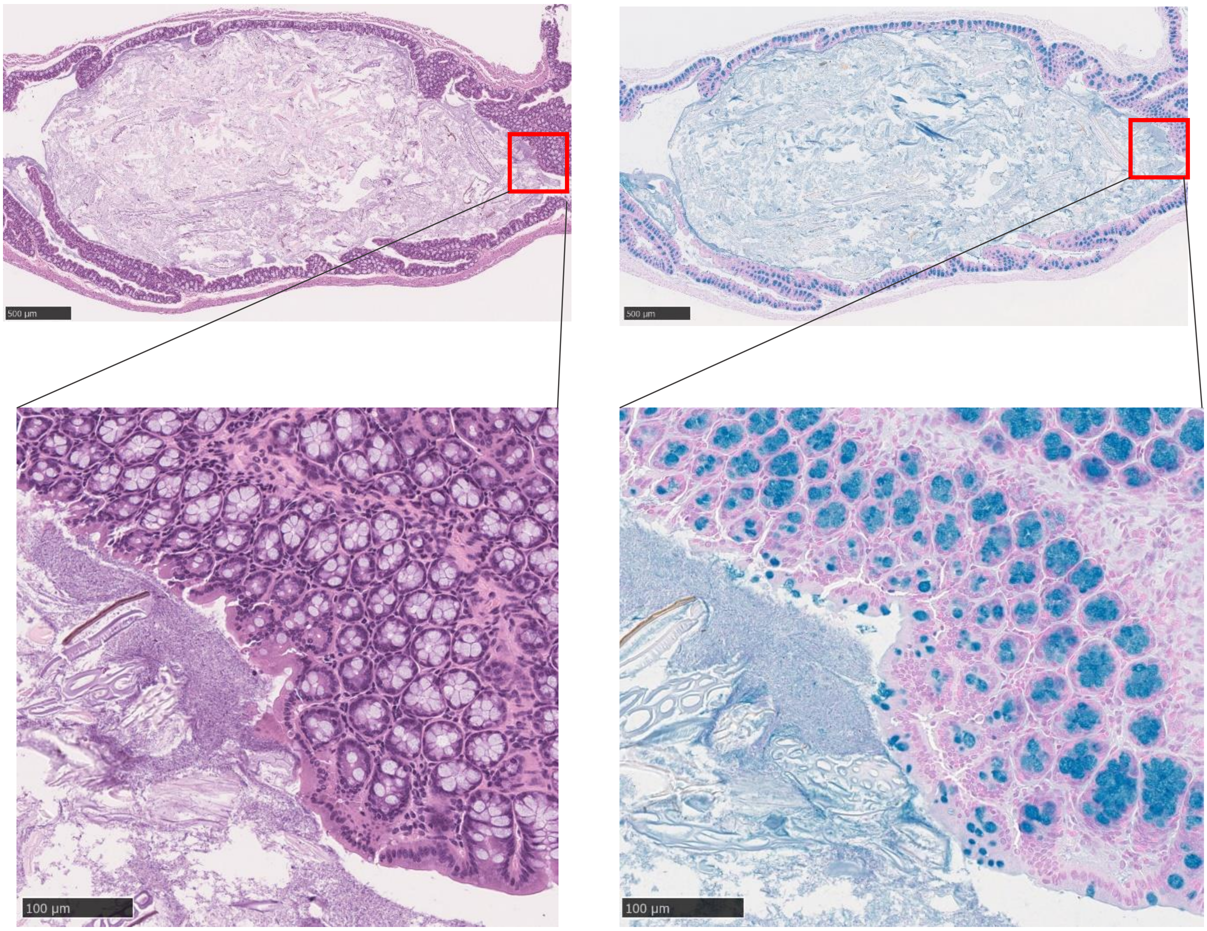

Figure S2: Histological staining of proximal colon samples from C57BL/6 mice.
(a) HE staining of longitudinal section of proximal colon.
(b) Alcian blue staining of longitudinal section of proximal colon.

Methods: Histological analysis was done by Genostaff Co.,Ltd. (Tokyo, Japan). Proximal colon samples used for histological analysis were fixed with 15% neutral formalin, embedded in paraffin, sectioned at 4 µm, and stained with hematoxylin and eosin Y (Sigma-Aldrich Co. LLC, Saint Louis, Missouri, USA) for HE staining and with alcian blue (Muto pure chemicals Co. Ltd, Tokyo, Japan) for alcian blue staining.
